# Associations between hospital characteristics, volume, and reasons for revision: a cohort study of 48,029 unicompartmental knee arthroplasties with 3,397 revisions from the Dutch Arthroplasty Register

**DOI:** 10.2340/17453674.2025.44961

**Published:** 2026-03-05

**Authors:** Hendrik W H DE RAADT, Iris KOENRAADT-VAN OOST, Anouk NIJS, Anneke SPEKENBRINK-SPOOREN, Leon ELMANS, Rutger C I VAN GEENEN

**Affiliations:** 1 Foundation for Orthopedic Research, Care & Education, Amphia Hospital, Breda; 2 Department of Orthopedic Surgery, Amphia Hospital, Breda; 3 Dutch Arthroplasty Register (LROI), ‘s-Hertogenbosch, the Netherlands

## Abstract

**Background and purpose:**

It remains debated whether high annual hospital volumes for unicompartmental knee arthroplasty (UKA) are associated with a low risk of revision, and what explanations may underlie this relationship. We aimed to analyze the association between specific hospital characteristics defined as volume, type, and referral for revision, and frequency and reasons for UKA revision.

**Methods:**

Data from primary UKAs (2007–2022) and their revisions were extracted from the Dutch Arthroplasty Register. Hospitals were categorized by type; academic, top clinical teaching, private, or other general hospitals. Hospitals were grouped by annual UKA volume: ≤24, 25–39, 40–79, and ≥80 procedures. Multiple linear regression assessed the relationship between the number of revisions with hospital volume and type, adjusted for confounders. Chi-squared tests were used to test for differences in revision reasons based on volume and referrals.

**Results:**

48,029 primary UKAs and 3,397 revisions were included. High-volume and top clinical teaching hospitals had a significantly lower risk of revision following primary UKA (P < 0.001). Cementless implants had a lower risk compared with cemented implants. Revision reasons varied by hospital volume and whether revision occurred after referral (P < 0.001). Loosening, progression of osteoarthritis, malalignment, and pain were less common in the highest volume hospitals. If revision occurred after referral, malalignment was more frequently registered as the reason for revision.

**Conclusion:**

High-volume and top clinical teaching hospitals were associated with lower risk of revision following primary UKA. Differences in revision reasons, with fewer cases of loosening, progression of osteoarthritis, malalignment, and pain, may explain the lower risk of revision at higher volume hospitals.

Unicompartmental knee arthroplasty (UKA) is associated with higher revision rates compared with total knee arthroplasty (TKA) [1-5]. Compared with TKA, the risk of revision after UKA in the Netherlands is approximately twice as high [1]. Gaining a deeper understanding of this increased risk is essential for improving patient outcomes.

Several factors contribute to the higher risk of revision observed in UKA. These include patient selection, prosthesis characteristics, and hospital volumes. For instance, deviation from recommended indications – such as performing UKA on patients with inflammatory disease, post-osteotomy valgus deformity, or partial thickness lesions – significantly increases the risk of revision [6]. Patient and prosthesis characteristics also play a role; younger patients, female patients, and those with cemented implants have a higher risk of revision [3, 7-12]. Furthermore, UKAs performed in low-volume hospitals are associated with increased risk of revision, suggesting that volume is a critical factor [3,7,12-17].

Hospital volume and whether patients were referred to another hospital may also influence the reasons for revision. Certain revisions, such as those for unexplained pain or loosening, may be potentially avoidable and might not be beneficial for patients [18,19]. These avoidable revisions could increase the overall risk of revision without improving outcomes. If these types of revisions occur more frequently in low-volume hospitals, this may partly explain the differences in the risk of revision between high- and low-volume hospitals. However, it remains unclear whether hospital volume or referral for revision affects the underlying reasons for revision.

To address this knowledge gap, our study examined the characteristics of UKA revisions. The aim of our study was to analyze the association of specific hospital characteristics defined as volume, type, and referral for revision, with frequency and reasons for revision.

## Methods

### Study design and population

This retrospective cohort study analyzed data from the Dutch Arthroplasty Register (LROI) for all primary and revision UKAs performed between 2007 and 2022. The Dutch Arthroplasty Register has had a completion rate of 97–100% for primary and revision knee arthroplasty since 2015 [20]. Revisions were defined as any insertion, removal, or replacement of 1 or more components of the prosthesis. Only first revisions after primary UKAs were included in the analysis. All data were anonymized prior to extraction from the register, stored securely, and accessible only to the investigators. This study is reported according to the STROBE guidelines.

### Data collection

Data on patient, prosthesis, and hospital characteristics were selected from the LROI.

Patient characteristics included age, sex, body mass index, smoking status, ASA classification, Charnley score, diagnosis, referral to another hospital before revision, reasons for revision, type of revision, and follow-up. Age was recorded at primary UKA (index surgery) and categorized into 5 groups: < 50, 50–59, 60– 69, 70–79, and ≥ 80 years. Types of revision were categorized as total revision (conversion to TKA), partial revision, or prosthesis removal. Follow-up was defined as the interval from the primary procedure to revision, death, or end of follow-up (January 1, 2023).

Prosthesis characteristics included the bearing type (fixed or mobile) and fixation method of the implants (cemented, uncemented, or hybrid). Hybrid fixation was defined as cemented fixation of the tibial component combined with uncemented fixation of the femoral component.

Hospital characteristics included annual absolute and proportional hospital volume and hospital type. Absolute hospital volume was defined as the number of primary UKAs performed per annum, categorized into quartiles: 1–24, 25–39, 40–79, and 80–330 UKAs. Proportional hospital volume was defined as the percentage of knee arthroplasties that were UKAs, calculated as UKA / (TKA + UKA) x 100%, and categorized into quartiles: ≤ 8.7%, 8.8–14.3%, 14.4–25.3%, and ≥ 25.4%. Hospital type was categorized into academic hospitals, private hospitals, top clinical teaching hospitals (STZ label), or other general hospitals. The collaboration of top clinical teaching hospitals (STZ) label is granted to 27 Dutch hospitals that meet specific quality criteria as top clinical teaching hospitals [20].

### Statistics

Missing data were omitted only from the analyses for which they were required. Patient, prosthesis, and hospital characteristics were assessed for normality and analyzed using descriptive statistics. The interquartile range (IQR) was calculated for variables that were not normally distributed. Variables showing differences between patients with and without UKA revisions were tested for statistical significance and 95% confidence intervals (CI) were calculated using Student’s t-test, ANOVA, or Pearson’s chi-square test, as appropriate.

To examine the association between hospital volume and type with frequency of revisions, multiple linear regression analysis was performed with the proportion of UKAs revised per hospital as the dependent variable and hospital volume and type as independent variables. Two models were used:

*Absolute hospital volume:* Percentage of revisions = 34.45 – 10.56 x log (Hospital volume) – 1.03 x (top clinical teaching hospital) – 0.07 x (Age) – 0.95 x (Charnley B1) – 0.84 x (Charnley B2) – 1.36 x (Cemented fixation) – 2.14 x (Hybrid).

This model was used to estimate the relationship between absolute hospital volume and revision percentages, controlling for hospital type. Model fit was assessed using the coefficient of determination (R² = 0.36) and overall significance (F(7, 1,730) = 136.4, P < 0.001).

*Proportional hospital volume*: Percentage of revisions = 24.37 – 0.21 × (Proportional hospital volume) – 2.99 × (Top clinical teaching hospital) – 0.09 × (Age) + 4.90 × (Academic hospital) – 1.00 × (Charnley B1) – 1.16 × (Charnley B2) – 1.97 × (Cemented fixation) – 3.55 × (Hybrid) – 1.24 × (Mobile bearing).

This model assessed the effect of proportional hospital volume on revision percentages. Model fit was evaluated using R² = 0.23 and F(9, 1,685) = 55.5, P < 0.001.

Both models initially included only hospital volume and type as independent variables. Subsequently, all patient and prosthesis characteristics were added as independent variables to adjust for possible confounding. Assumptions of linearity and normality were checked, and heteroscedasticity was addressed by applying a logarithmic transformation to hospital volume. Associations between hospital volume and reasons for revision were analyzed using cross-tabulation with Pearson’s chi-square test. P < 0.05 was considered statistically significant. All statistical analyses were performed using the statistical package SPSS (version 29, IBM Corp, Armonk, NY, USA).

### Ethics, funding, data sharing, AI tools, and disclosures

Ethical approval was not required, as the study was based on anonymized registry data. The study received no funding. Due to data protection agreements and legal restrictions, the de-identified registry data used in this study cannot be shared. Data were stored locally in a password-encrypted environment and will be deleted after study completion. During the preparation of this manuscript, grammar corrections and sentence refinements were performed using OpenAI’s ChatGPT (version GPT-4.0). The authors declare no conflicts of interest. Complete disclosure of interest forms according to ICMJE are available on the article page, doi: 10.2340/17453674.2025.44961

## Results

**Figure 1:**
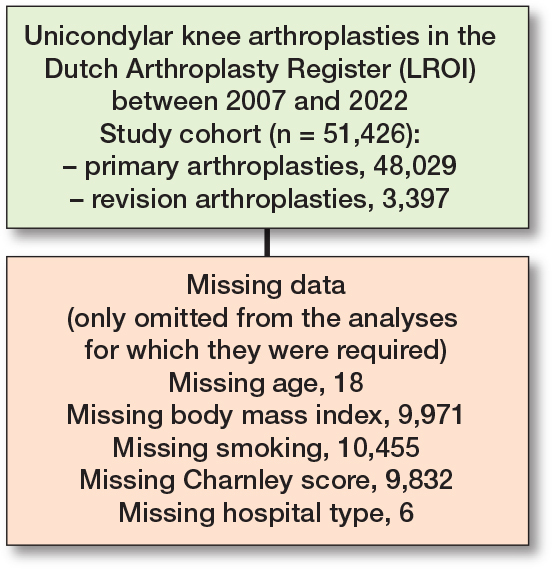
Patient flowchart.

48,029 primary UKAs and 3,397 revision UKAs were performed during the study period (Figure 1). There were no patient exclusions. However, some variables had missing data in the register. BMI, smoking status, and charnley score were not registered before 2014, which explains the substantial proportion of missing values.

### Patient, prosthesis, and revision characteristics

Younger age, female sex, higher body mass index, smoking, a lower ASA classification, and a lower Charnley score were significantly associated with the revised UKA group (Tables 1 and 2). Patient and prosthesis characteristics, stratified by quartiles based on annual absolute hospital volume, can be found in Table S1 (see Supplementary data).

**Table 1:** Patient and prosthesis characteristics at the time of primary UKA. Values are count (%) unless otherwise specified

Factor	Revised UKA (n = 3,397)	Unrevised UKA (n = 44,632)	P value	Difference mean (CI)
Age, mean (SD)	60.8 (8.8)	64.2 (8.8)	<0.001	–3.4 (–3.7 to –3.1)
Female	1,990 (59)	24,844 (55)	<0.001	
BMI, mean (SD)	29.3 (4.5)	29.0 (4.4)	0.007	0.3 (0.1 to 0.5)
Smoker	227 (13)	3,305 (9.2)	<0.001	
ASA			<0.001	
I	1,046 (31)	10,278 (23)		
II	1,908 (56)	28,170 (63)		
III–IV	286 (8.4)	5,365 (12)		
Charnley			<0.001	
A	1,095 (60)	18,543 (51)		
B1	471 (26)	11,199 (31)		
B2	241 (13)	6,086 (17)		
C	23 (1.3)	539 (1.5)		
Diagnosis			0.03	
Osteoarthritis	3,318 (99)	43,732 (99)		
Osteonecrosis	19 (0.6)	389 (0.9)		
Late post-traumatic	14 (0.4)	171 (0.4)		
Rheumatoid arthritis	4 (0.1)	34 (0.1)		
Other	11 (0.3)	66 (0.1)		
Follow-up, median	2.0	4.1		
years, IQR	0.9–4.6	1.7–7.5	<0.001	
Bearing type			<0.001	
Mobile	2,536 (78)	33,638 (81)		
Fixed	700 (22)	7,997 (19)		
Fixation			<0.001	
Cemented	2,334 (70)	22,677 (51)		
Cementless	925 (28)	20,723 (47)		
Hybrid	76 (2.3)	793 (1.8)		

CI: 95% confidence interval, IQR: interquartile range, SD: standard deviation

**Table 2:** Revision characteristics. Values are count (%)

Factor	Revised UKA (n = 3,397)
Revision in another hospital	648 (19)
Reason for revision	
Progression of osteoarthritis	558 (19)
Loosening	527 (18)
Instability	388 (13)
Malalignment	298 (10)
Patellar pain	261 (8.7)
Infection	186 (6.2)
Wear inlay	146 (4.9)
Periprosthetic fracture	109 (3.6)
Unspecified removal	51 (1.7)
Bearing dislocation	39 (1.3)
Patellar dislocation	16 (0.5)
Pain and wear inlay	11 (0.4)
Arthrofibrosis	10 (0.3)
Patellar dislocation and wear inlay	1 (0.0)
Other	393 (13)
Revision type	
Total revision	2,479 (73)
Partial revision	842 (25)
Partial revision, only insert or patella	745 (22)
Removal of prosthesis	42 (1.2)
Patella addition	8 (0.2)
Other	8 (0.2)

“Unspecified removal” was defined as explantation of the primary UKA followed by re-implantation of a knee prosthesis without a specified explanation.

Patients who underwent revision had a shorter median follow-up (2.0 years, IQR 0.9–4.6) compared with non-revised patients (4.1 years, IQR 1.7–7.5). In 70% of patients who underwent revision, the primary implant used was a cemented UKA, compared with 51% in patients with an unrevised UKA (unadjusted comparison, P < 0.001). Fixed-bearing implants were slightly more common among revised UKAs than unrevised UKAs (21% vs 19%, P < 0.001). The most frequent reasons for revision were progression of osteoarthritis and implant loosening. Additionally, 19% of revision UKAs occurred after referral to a different hospital.

### Hospital characteristics

Details of hospital characteristics are provided in Table 3. Most revision surgeries were carried out in general hospitals and top clinical teaching hospitals, while hospital volumes for primary UKA were highest in private hospitals.

**Table 3:** Hospital characteristics during the study period (2007–2022). Values are count (row wise %) or as specified

Factor	All hospitals combined	Academic hospital	Private hospital	Top clinical teaching hospital	Other general hospital
Number of hospitals	102	6 (5.9)	22 (22)	28 (28)	43 (42)
Primary UKAs	48,029	544 (11)	11,509 (24)	18,684 (39)	17,286 (36)
Revision UKAs	3,397	59 (1.7)	607 (18)	1,297 (38)	1,434 (42)
Number of revision UKAs during study period, mean	33	10	28	46	33
Annual primary UKA hospital volume					
Absolute number, mean (SD)	61.6 (59.1)	12.4 (8.0)	107.4 (84.0)	64.8 (53.9)	41.2 (35.4)
Proportion, mean % (SD)	17.8 (12.4)	10.5 (5.5)	29.3 (14.3)	15.2 (9.8)	15.7 (11.0)

### Associations between hospital volume and revision percentage

Higher absolute and proportional hospital volumes were associated with lower revision percentages. In the absolute hospital volume model (P < 0.001), each tenfold increase in hospital volume was associated with a 10.6% decrease in the revision percentage (P < 0.001). Using the median values of the volume quartiles (16, 32, 55, and 145 UKAs/year), the corresponding predicted revision percentages decreased across quartiles (approximately 21.7%, 18.6%, 16.1%, and 11.6%, respectively), demonstrating a clear volume-gradient effect. Although the model overestimated the absolute percentages relative to the observed overall revision rate, the predicted trend showed lower revision percentages in higher-volume hospitals. Top clinical teaching hospitals also showed lower predicted revision percentages compared with other hospitals (P < 0.001).

In the proportional hospital volume model (P < 0.001), higher proportional UKA volume was associated with lower revision percentages (P < 0.001). Using the median values of the proportional volume quartiles (8.0%, 14.9%, 25.5%, and 38.1%), the corresponding predicted revision percentages decreased across quartiles (approximately 22.7%, 21.2%, 19.0%, and 16.4%, respectively). Although the model overestimated the absolute percentages relative to the observed revision rate, the predicted values demonstrated a clear volume-gradient effect. Top clinical teaching hospitals again showed lower predicted revision percentages compared with other hospitals (P < 0.001).

**Figure 2:**
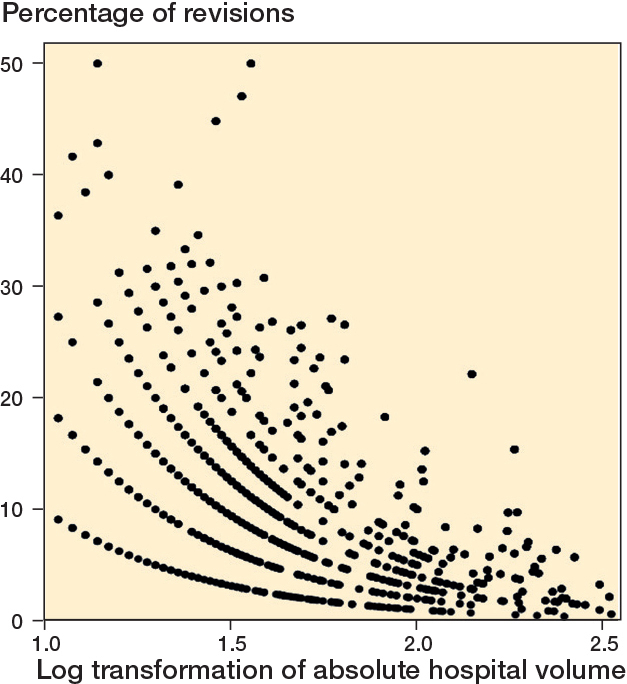
Scatterplot of log transformation of absolute hospital volume versus percentage of revisions.

**Figure 3:**
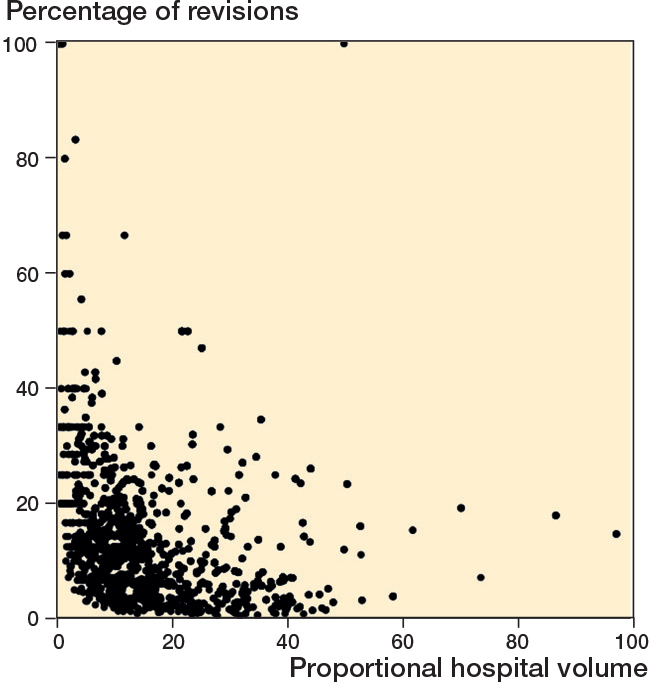
Scatterplot of proportional hospital volume versus percentage of revisions.

Visual representations of the relationships between log-transformed absolute hospital volume, proportional hospital volume, and revision percentages are shown in Figures 2 and 3.

### Reasons for revision

#### By absolute hospital volume

Reasons for revision differed significantly across hospital volume groups (P < 0.001; Table S2, see Supplementary data). These comparisons were unadjusted and reflect observed differences in revision indications across hospital volume quartiles. In the lowest volume group (1–24 UKAs), loosening was the most frequent reason for revision, whereas instability and infection were less common. Progression of osteoarthritis was predominant in mid-volume groups (25–39 and 40–79 UKAs). In the highest volume group (80–330 UKAs), most revisions were performed for instability, and in lower volume hospitals (1–24, 25–39, and 40–79 UKAs), the most frequent reasons for revision were both instability and infection. Additionally, loosening, progression of osteoarthritis, malalignment, and pain were less common in the highest volume group (80–330 UKAs) compared with lower volume groups (1–24, 25–39, and 40–79 UKAs).

#### By proportional hospital volume

Significant differences in reasons for revision were also observed across proportional hospital volume groups (P < 0.001; Table S3, see Supplementary data). These comparisons were unadjusted and represent observed differences in revision indications across proportional hospital volume quartiles. Loosening was the most frequent reason for revision in the lowest volume group (≤ 8.7%), along with malalignment and progression of osteoarthritis. These reasons were more prevalent than in the highest volume groups (8.8–14.3%, 14.4–25.3%, and ≥ 25.4%). In mid-proportional volume groups (8.8–14.3% and 14.4–25.3%), progression of osteoarthritis was the most common reason. In the highest volume group (≥ 25.4%), other causes were the most common reason for revision, and instability and infection were observed more frequently than in lower-proportional volume groups (≤ 8.7%, 8.8–14.3%, 14.4–25.3%).

### Referral status

Revisions performed after referral to another hospital were more often due to malalignment (19.6% after referral vs. 7.7% without referral; P < 0.001). Other reasons for revision did not significantly differ based on referral status.

## Discussion

The aim of our study was to analyze the association of specific hospital characteristics, such as volume, type, and referral for revision, with frequency and reasons for revision. One of our key findings is that the risk of revision was lower in high-volume UKA hospitals and top clinical teaching hospitals, even after adjusting for confounding variables. Secondly, the reasons for revision showed variation based on hospital volume, with fewer revisions in high-volume hospitals due to loosening, progression of osteoarthritis, malalignment, and pain compared with low-volume hospitals. Finally, we found that cemented implants have a higher risk of revision.

We observed significant differences in patient and prosthesis characteristics between patients with revised and unrevised UKAs, though not all were clinically relevant. Notable findings include that patients undergoing revision surgeries were more likely to have an ASA I classification, a Charnley A score, and cemented implants at the time of primary UKA. Not in line with previous studies is the finding that more ASA I patients underwent revision, possibly due to surgeons avoiding surgery in higher risk (ASA II–IV) patients [7]. The larger proportion of Charnley A scores may be explained in the same way. In line with previous studies, we found that cemented implants were at higher risk of revision, due to higher rates of aseptic loosening [10, 21]. Cemented implants did not show lower rates of periprosthetic fracture, although such a difference might be expected. This finding may be influenced by incomplete registration of fractures as a complication within the registry. In line with previous studies, patients undergoing revision surgeries were more likely to have a younger age, female sex, higher BMI, and to be smokers at primary UKA [11]. Although fixed-bearing implants were statistically more common among revised UKAs, the absolute difference was small and of uncertain clinical relevance.

In our regression models, higher absolute and proportional hospital volumes were associated with a lower risk of revision UKA, supporting previous findings [12,17]. A new observation is that hospital type was also associated with risk of revision, regardless of hospital volume. Specifically, top clinical teaching hospitals demonstrated lower percentages of revision UKA compared with other hospital types. A possible explanation is that, in these hospitals, surgical indications undergo routine peer review before being approved for surgery for quality of care and educational purposes. This routine work-up may largely eliminate incorrect indications for primary and revision surgery. A similar approach is also applied in academic hospitals for educational purposes, but these hospitals generally perform fewer UKAs. As hospital volume is a critical factor for outcome and expertise, this difference in hospital volume may contribute to the higher revision risk observed in academic hospitals compared with top clinical teaching hospitals. However, this explanation remains highly speculative.

Among the highest volume hospitals, less frequently registered reasons for revision included loosening, progression of osteoarthritis, malalignment, and pain. One possible explanation for the lower prevalence of loosening in high-volume hospitals is that increased surgical exposure may help to recognize and correctly interpret physiological radiolucent lines beneath the components from true loosening. Physiological radiolucent lines should not be considered an indication for revision in patients experiencing pain, as they are generally not associated with symptomatic discomfort [22]. Moreover, these lines are mostly observed in cemented implants, which tend to be revised more frequently, offering further plausibility to this explanation [21].

Progression of osteoarthritis may result from medial compartment overstuffing, pre-existing valgus alignment, underlying inflammatory disease, or undetected cartilage damage in the lateral compartment during primary surgery [23-25]. To achieve high volumes, surgeons should adhere to well-defined indications and systematic preoperative evaluations. This approach likely reduces errors in patient selection, which may account for the lower proportion of revisions attributed to the progression of osteoarthritis in high-volume hospitals.

In contrast, the lowest volume hospitals showed higher rates of malalignment and patellar pain as reasons for revision. This finding is consistent with earlier studies from the Nordic countries, which reported a higher incidence of pain as a reason for revision in low-volume hospitals [14]. However, in our study, the percentage of revisions for patellar pain in the highest volume hospitals was only marginally lower. Variations in the labelling of knee pain following arthroplasty may account for the discrepancies between our findings and those of other studies. For instance, in the Dutch arthroplasty register, knee pain is categorized as patellar pain, whereas in the Nordic countries it is generally labelled simply as “pain”.

Revisions following referral to another hospital were more often performed for malalignment. A possible explanation is that surgeons may be less critical of their own procedures. Additionally, the diagnosis malalignment is challenging; it requires well-aligned, preferably full-leg, radiographs to accurately assess varus or valgus alignment [26]. Similarly, malposition is classified as malalignment and requires well aligned or fluoroscopically guided radiographs to determine whether deviations are within or outside the safe range [26]. High-volume centers are more likely to gain expertise in obtaining and interpreting specific diagnostic modalities. Furthermore, patients referred for revision are more likely to be treated at high-volume centers, potentially explaining the higher proportion of malalignment diagnoses.

### Limitations

The registry-based nature of the data limited the ability to validate the reasons for revision and prevented the study from proving causal relationships. Additionally, the “other reasons” category was among the most prevalent reasons for revision in the highest volume hospitals, yet specific details regarding this category were not available. It might be of interest to clarify what the other reasons entail in future research. Moreover, residual confounding may exist in our regression models due to reliance on registry variables.

Our study focused exclusively on hospital volumes, specifically examining annual volumes, and did not account for the nationwide increase in absolute and proportional volumes. This trend may further enhance quality and potentially influence revision rates and the reasons for revision.

### Conclusion

We found that high-volume hospitals were associated with a lower risk of revision for UKA. We also found that certain hospital types, specifically top clinical teaching hospitals, were associated with a lower risk of revision. Moreover, high-volume hospitals differed in their reasons for revision compared with low-volume hospitals, showing fewer cases of loosening, progression of osteoarthritis, malalignment, and pain. These findings may help to understand how hospital volume influences the risk of revision in UKA.

### Supplementary data

Tables S1–S3 are available as supplementary data on the article page, doi: 10.2340/17453674.2025.44961

## Supplementary Material



## Notes

RG and IK initiated the study. HR performed the statistical analyses and wrote the initial manuscript. All authors contributed to the interpretation of the results, participated in manuscript revisions, and accepted the final version of the manuscript.

Handling co-editor: Per Henrik Randsborg

*Acta* thanks Karin Svensson Malchau and Anders Troelsen for help with peer review of this manuscript.
