# Supplementary material for: Associations between hospital characteristics, volume, and reasons for revision: a cohort study of 48,029 unicompartmental knee arthroplasties with 3,397 revisions from the Dutch Arthroplasty Register
Source: Acta Orthop. 2026 Mar 5;97:164–70. doi: 10.2340/17453674.2025.44961 (PMC12963959; doi:10.2340/17453674.2025.44961)
Supplement: Supplementary file 1 [file ActaO-97-44961-s1.pdf]

# Supplementary data

**Table S1. Patient and prosthesis characteristics at the time of primary UKA. Values are count (%) unless otherwise specified**

| | Absolute annual hospital volume | | | |
|---|---|---|---|---|
| | **<25 (n = 6,788)** | **25–39 (n = 7,582)** | **40–79 (n = 11,551)** | **>79 (n = 22,108)** |
| Age, mean (SD) | 61.7 (8.5) | 63.0 (8.8) | 64.0 (8.9) | 64.9 (8.8) |
| Female | 3,940 (58) | 4,258 (56) | 6,427 (56) | 12,209 (55) |
| Body mass index, mean (SD) | 29.2 (4.4) | 29.1 (4.4) | 29.1 (4.4) | 29.0 (4.5) |
| Smoker | 424 (12) | 514 (12) | 889 (9.8) | 1,705 (8.3) |
| ASA | | | | |
| *I* | 2,113 (31) | 2,048 (27) | 2,691 (23) | 4,472 (20) |
| *II* | 3,760 (55) | 4,465 (59) | 7,347 (64) | 14,506 (66) |
| *III–IV* | 580 (8.5) | 717 (9.5) | 1,382 (12) | 2,972 (13) |
| Charnley | | | | |
| *A* | 2,018 (57) | 2,518 (57) | 5,038 (54) | 10,064 (48) |
| *B1* | 1,027 (29) | 1,212 (27) | 2,759 (30) | 6,672 (32) |
| *B2* | 472 (13) | 669 (15) | 1430 (15) | 3,756 (18) |
| *C* | 37 (1.0) | 42 (0.9) | 111 (1.2) | 372 (1.8) |
| Diagnosis | | | | |
| *Osteoarthritis* | 6,581 (98) | 7,403 (98) | 11,306 (99) | 21,760 (99) |
| *Osteonecrosis* | 45 (0.7) | 58 (0.8) | 111 (1.0) | 194 (0.9) |
| *Late post-traumatic* | 39 (0.6) | 37 (0.5) | 39 (0.3) | 70 (0.3) |
| *Rheumatoid arthritis* | 4 (0.1) | 10 (0.1) | 12 (0.1) | 12 (0.1) |
| *Other* | 29 (0.4) | 16 (0.2) | 13 (0.1) | 19 (0.1) |
| Follow-up in years, median (IQR) | 7.7 (4.2–11.2) | 6.7 (3.2–10.7) | 3.8 (1.7–7.1) | 2.6 (1.0–5.0) |
| Bearing type | | | | |
| *Mobile* | 4,359 (67) | 5,905 (81) | 9,382 (85) | 16,528 (83) |
| *Fixed* | 2,174 (33) | 1,347 (19) | 1,660 (15) | 3,516 (17) |
| Fixation | | | | |
| *Cemented* | 5,487 (82) | 5,258 (71) | 6,044 (53) | 8,222 (37) |

| | | | | |
|---|---|---|---|---|
| *Cementless* | 1,121 (17) | 2,051 (28) | 5,261 (46) | 13,215 (60) |
| *Hybrid* | 64 (1.0) | 126 (1.7) | 152 (1.3) | 527 (2.4) |

**Table S2. Reasons for revision compared between different absolute hospital volumes**

| | Absolute hospital volume groups | | | |
|---|---|---|---|---|
| | <25 (n = 870) % (rank) | 25–39 (n = 873) % (rank) | 40–79 (n = 823) % (rank) | >79 (n = 831) % (rank) |
| *Progression of osteoarthritis* | 22 (2) | 23 (1) | 17 (1) | 13 (3) |
| *Loosening* | 26 (1) | 19 (2) | 16 (3) | 9.8 (5) |
| *Instability* | 9.0 (5) | 9.1 (6) | 16 (2) | 18 (1) |
| *Malalignment* | 12 (3) | 11 (4) | 8.5 (6) | 7.7 (6) |
| *Patellar pain* | 9.8 (4) | 10 (5) | 8.9 (5) | 6.1 (8) |
| *Infection* | 3.3 (8) | 4.0 (8) | 6.1 (7) | 12(4) |
| *Wear inlay* | 4.6 (7) | 5.5 (7) | 5.8 (8) | 3.5 (9) |
| *Periprosthetic fracture* | 1.9 (9) | 2.1 (9) | 4.1 (9) | 6.5 (7) |
| *Unspecified removal* | 1.1 (10) | 2.0 (10) | 2.0 (10) | 1.8 (11) |
| *Bearing dislocation* | 0.3 (12) | 0.5 (11) | 2.0 (10) | 2.4 (10) |
| *Patellar dislocation* | 0.3 (12) | 0.3 (13) | 0.7 (12) | 0.9 (12) |
| *Pain and wear inlay* | 0.7 (11) | 0.4 (12) | 0.1 (14) | 0.3 (14) |
| *Arthrofibrosis* | 0.0 (15) | 0.3 (13) | 0.4 (13) | 0.7 (13) |
| *Patellar dislocation and wear inlay* | 0.1 (14) | 0.0 (15) | 0.0 (15) | 0.0 (15) |
| *Other* | 8.6 (6) | 13 (3) | 13 (4) | 18 (2) |

Multiple reasons may be recorded for each revision; therefore, cumulative percentages can exceed 100%.

"Unspecified removal" was defined as explantation of the primary UKA followed by re-implantation of a knee prosthesis without a specified explanation.

**Table S3. Reasons for revision compared between different proportional hospital volumes**

| | Proportional hospital volume groups | | | |
|---|---|---|---|---|
| | **<8.8% (n = 849) % (rank)** | **8.8–14.3% (n = 849) % (rank)** | **14.4–25.4% (n = 853) % (rank)** | **>25.4% (n = 846) % (rank)** |
| *Progression of osteoarthritis* | 21 (2) | 22 (1) | 17 (1) | 15 (3) |
| *Loosening* | 25 (1) | 19 (2) | 15 (3) | 12 (4) |
| *Instability* | 9.6 (5) | 13 (3) | 13 (4) | 16 (2) |
| *Malalignment* | 13 (3) | 10 (4) | 9.5 (5) | 7.5 (7) |
| *Patellar pain* | 8.0 (6) | 9.8 (6) | 9.4 (6) | 7.6 (6) |
| *Infection* | 4.2 (8) | 4.3 (8) | 6.9 (7) | 9.3 (5) |
| *Wear inlay* | 4.6 (7) | 5.9 (7) | 3.7 (9) | 5.2 (8) |
| *Periprosthetic fracture* | 2.5 (9) | 2.5 (9) | 4.2 (8) | 5.2 (8) |
| *Unspecified removal* | 1.4 (10) | 0.9 (10) | 1.3 (11) | 3.1 (10) |
| *Bearing dislocation* | 0.3 (13) | 0.7 (11) | 2.3 (10) | 1.3 (11) |
| *Patellar dislocation* | 0.6 (11) | 0.0 (14) | 1.1 (12) | 0.5 (12) |
| *Pain and wear inlay* | 0.4 (12) | 0.5 (12) | 0.1 (14) | 0.4 (14) |
| *Arthrofibrosis* | 0.0 (15) | 0.4 (13) | 0.4 (13) | 0.5 (12) |
| *Patellar dislocation and wear inlay* | 0.1 (14) | 0.0 (14) | 0.0 (15) | 0.0 (15) |
| *Other* | 10 (4) | 10 (5) | 16 (2) | 16 (1) |

Multiple reasons may be recorded for each revision; therefore, cumulative percentages can exceed 100%.

“Unspecified removal” was defined as explantation of the primary UKA followed by re-implantation of a knee prosthesis without a specified explanation.
